# Ecological comparison of six countries in two waves of COVID-19

**DOI:** 10.3389/fpubh.2024.1277457

**Published:** 2024-02-28

**Authors:** Meiheng Liu, Leiyu Shi, Manfei Yang, Jun Jiao, Junyan Yang, Mengyuan Ma, Wanzhen Xie, Gang Sun

**Affiliations:** ^1^Department of Health Management, School of Health Management, Southern Medical University, Guangzhou, China; ^2^Department of Health Policy and Management, Bloomberg School of Public Health, Johns Hopkins University, Baltimore, MD, United States

**Keywords:** COVID-19, non-drug interventions, vaccination measures, containment strategies, mitigation strategies, ecological comparison

## Abstract

**Objective:**

The purpose of this study is to provide experience and evidence support for countries to deal with similar public health emergencies such as COVID-19 by comparing and analyzing the measures taken by six countries in epidemic prevention and control.

**Methods:**

This study extracted public data on COVID-19 from the official website of various countries and used ecological comparative research methods to compare the specific situation of indicators such as daily tests per thousand people, stringency index, and total vaccinations per hundred people in countries.

**Results:**

The cumulative death toll in China, Germany and Australia was significantly lower than that in the United States, South Africa and Italy. Expanding the scale of testing has helped control the spread of the epidemic to some extent. When the epidemic situation is severe, the stringency index increases, and when the epidemic situation tends to ease, the stringency index decreases. Increased vaccination rates, while helping to build an immune barrier, still need to be used in conjunction with non-drug interventions.

**Conclusion:**

The implementation of non-drug interventions and vaccine measures greatly affected the epidemic prevention and control effect. In responding to public health emergencies such as the COVID-19 epidemic, countries should draw on international experience, closely align with their national conditions, follow the laws of epidemiology, actively take non-drug intervention measures, and vigorously promote vaccine research and development and vaccination.

## 1 Introduction

Since December 2019, a number of cases of pneumonia of unknown cause with a history of exposure to seafood markets in South China have been found in some hospitals in Hubei Province, China, which have been confirmed as acute respiratory infectious diseases caused by novel coronavirus infection. The outbreak has since rapidly spread around the world. The initial genome sequencing data of the virus did not match that of previously sequenced coronaviruses (1). On 30 January 2020, the World Health Organization declared the COVID-19 outbreak a public health emergency of international concern. The COVID-19 epidemic is characterized by high prevalence and long incubation period. According to official reports, as of December 31, 2022, the cumulative number of confirmed cases worldwide exceeded 700 million and the cumulative death toll exceeded 6.7 million (2). Due to the emergence of the mutated strain, countries have limited epidemic prevention and control measures, and the epidemic has rebounded several times, posing a serious threat to human life and seriously affecting global public health and economy (3, 4). And the emergence of mutated strains further compounds the challenge of containing the COVID-19 pandemic, as these mutants become more resistant to vaccines (5). The COVID-19 pandemic is considered the most serious public health threat since the 1918 H1N1 Spanish flu. The COVID-19 pandemic has had a severe impact on the economy, politics and people’s lives around the world, and has exposed the chronic deficiencies in the health systems of many countries (6). Based on this, some international scholars have also carried out extensive research on the status quo, impact and countermeasures of the epidemic. For example, some scholars have conducted in-depth studies on housing and public space during the COVID-19 epidemic and the impact of COVID-19 on urban public space (7, 8).

Non-drug interventions are actions taken by individuals and groups, other than vaccination and medication, to slow the spread of an epidemic disease, with the primary objective of controlling the source of infection and cutting off transmission routes. Vaccination is one of the effective methods to control the COVID-19 epidemic (9). It is therefore essential that countries set strategic targets for vaccine research and development and reach broad consensus (10). Since the outbreak of the epidemic, countries have taken non-drug intervention measures of different degrees and vaccine research and development and vaccination measures to control the spread of the epidemic according to the development status of the epidemic, economic strength, service capacity of the medical and health system, population distribution and other factors, in an attempt to control the large-scale spread of the epidemic from the perspective of controlling the source of infection, cutting off the route of transmission, and protecting the vulnerable population (11). With the development and spread of the epidemic, different countries have achieved different results in the prevention and control efforts. In this study, six countries including China, Germany, Australia, the United States, South Africa and Italy were selected as research objects. China was the first country to detect the novel coronavirus, and has controlled the rapid spread of the epidemic in a relatively short period of time, so it is necessary to conduct an in-depth analysis of its successful experience. Germany and Australia are representative countries in Europe and Oceania respectively, and have their own characteristics in epidemic management. The United States, South Africa and Italy have all been hit hard by the outbreak, and have been among the hardest hit countries in the global epidemic. At the same time, the selection of these countries also takes into account the relatively even geographical distribution. Based on the implementation intensity of non-drug intervention measures and the implementation of vaccination, this study analyzed the strategies and effects of epidemic prevention and control in each country. In view of the heavy losses caused by the COVID-19 epidemic to mankind, the international community has paid close attention to the research related to public health emergencies. To a certain extent, this study provides a small reference for the management of public health emergencies.

## 2 Research objects and methods

### 2.1 Research objects and indicators

Six typical countries, namely China in Asia, Germany and Italy in Europe, Australia in Oceania, the United States in North America, and South Africa in Africa, were selected as research objects. Indicators such as total cases and deaths per million people, daily tests per thousand people, stringency index, total vaccinations per hundred people, daily new cases per million people, and daily new deaths per million people were included in the analysis.

### 2.2 Data sources

In this study, official Data of countries on COVID-19 were collected from the official website of the World Health Organization and the Novel Coronavirus Resource Center of Johns Hopkins University, specific measures taken in response to COVID-19 were collected from government websites of countries, and indicator data involved in this study were collected from Our World in Data website (12–20). The purpose of the World Health Organization is to achieve the highest possible level of health for the world’s people. During the COVID-19 epidemic, the World Health Organization updated authoritative data daily, which provided the data source for this study. The Novel Coronavirus Resource Center of Johns Hopkins University has provided unprecedented near-real-time data tracking of this unprecedented outbreak, providing visualizations of reported cases and deaths to the public, journalists and policymakers around the world. Our World in Data is a website that provides global data. The research team of Our World in data is affiliated with the Oxford Martin Global Development Program at the University of Oxford in the United Kingdom, and focuses on decades of national data on human living standards. The data density and value of the website are high, including population, economic, health and other data, providing us with a comprehensive perspective of global population and health. The Oxford Coronavirus Government Response Tracker (OxCGRT) project calculate a Stringency Index, a composite measure of nine of the response metrics. Nine metrics are used to calculate the Stringency Index: school closures; workplace closures; cancellation of public events; restrictions on public gatherings; closures of public transport; stay-at-home requirements; public information campaigns; restrictions on internal movements; and international travel controls. The index on any given day is calculated as the mean score of the nine metrics, each taking a value between 0 and 100. A higher score indicates a stricter response (100 = strictest response). Data collection varies from country to country; for example, Germany does not continuously report the number of tests. In order to improve the visibility and accuracy of the results, the index of “new tests per thousand” in this study uses the smoothed data after official processing on the database website.

### 2.3 Research methods

Ecological comparative study is a method widely used in ecological research. The simplest method is to observe the distribution of a disease in different populations or regions, and then propose etiological hypotheses based on the differences in the distribution of the disease. Ecological comparative research can also be applied to evaluate the effectiveness of social facilities, population interventions, and the implementation of policies and laws. This study uses ecological comparative study method to describe the epidemic prevention policies and vaccine measures in six countries. In this study, Excel data analysis software is used to make visual analysis by making statistical charts. Based on the analysis of the epidemic prevention and control effect in six countries, this study proposed the hypothesis that “the implementation of non-drug interventions and vaccine measures greatly affected the epidemic prevention and control effect.” The effects of non-drug interventions and vaccination on epidemic prevention and control in each country were analyzed by comparing the indicators such as total cases and deaths per million people, daily tests per thousand people, stringency index, and total vaccinations per hundred people, combined with the development status of the epidemic in each country, the economic strength, the service capacity of the medical and health system, and the population distribution.

## 3 Results

### 3.1 Non-drug interventions by countries

#### 3.1.1 Containment strategies of China, Germany, and Australia

China, Germany, and Australia have tended to adopt strict containment strategies in the fight against COVID-19. China is following a classic containment strategy. Rapid response is critical in the early stages of an infectious disease outbreak (21). China became the first country to report a case of pneumonia of unknown cause after it was first reported in Wuhan, Hubei province. As the epidemic spread, the Chinese government attached great importance to it, took swift measures and brought the epidemic under control in a relatively short period of time (22, 23). During the epidemic, major measures include establishing a command system for COVID-19 prevention and control and building medical isolation facilities. When the epidemic is spreading, communities should play a role in epidemic prevention by adopting lockdown and medical treatment policies. In the phase of regular epidemic prevention and control, targeted measures have been taken across the country at different regions and levels to prevent imports and rebound at home. The policy focus on COVID-19 has shifted from medical support in the early stage to economic development in the later stage (24). Germany, one of the early countries in Europe to be affected by the outbreak, confirmed its first case of the novel coronavirus on January 27, 2020 local time, with an explosive increase in confirmed cases in late February. The German government has taken various measures to limit the gathering of people, strictly prevent the importation of the virus from abroad, initiated emergency plans, and increased the number of medical staff and hospital beds in an effort to contain the spread of the epidemic. Germany and Hong Kong produced the WHO-approved diagnostic test kits and distributed them to countries around the world on January 17, 2020. In addition, affected by the novel coronavirus epidemic, the German economy shrank and the employment situation was grim. The German government implemented the “short-time working plan.” Germany has a wide distribution of laboratories qualified for virus testing, and timely inclusion of relevant costs in the medical reimbursement system. Strict public health policies and generous social policies have been successful in Germany (25). Among the many countries affected by the novel coronavirus, Oceania’s epidemic prevention and control effect is relatively optimistic. Australia is relatively isolated on the land, and most of the domestic outbreaks are imported from abroad and spread in clusters. Since January 2020, Australia has screened airline passengers and subsequently ordered the closure of all public commercial places to prevent the spread of the virus. Unprecedented government spending on health care, employment and housing may have reduced anxiety and stress among some Australians (26). Although the worst impact was avoided, it still suffered many negative effects (27, 28). (Examples of specific measures taken by China, Germany and Australia are shown in Table 1).

**Table 1:** Major non-drug interventions in response to COVID-19 in China, Germany, and Australia.

Measure/Country	China	Germany	Australia
Overall strategy	Containment strategy		
Government response	The National Health Commission has included the novel coronavirus as a Class B infectious disease under the Law on the Prevention and Treatment of Infectious Diseases and implemented Class A management. Led by the National Health Commission, a joint prevention and control mechanism involving more than 30 departments has been established.	The Ministry of Health and the Ministry of the Interior announced the establishment of a federal outbreak response headquarters. The level of risk assessment for COVID-19 has been improved and a series of prevention and control measures have been developed based on the assessment of the German Federal Centre for Disease Control.	Australian Prime Minister Morrison has announced the launch of a COVID-19 emergency response plan. The premier of Victoria has declared a level two alert in Melbourne at a press conference. The Victorian government has upgraded its state of emergency to a “state of disaster.”
Community policy	Thirty provinces, autonomous regions and municipalities have formulated and implemented community-based prevention and control measures. Community publicity prevention common sense and actively guide response, eliminate panic.	Postponed or cancelled community public events. Germany has made it mandatory for people to wear face masks when travelling.	Enforced strict social distancing rules; Maintain a social distance of every 4 square meters/person, and 1.5 meters was recommended for outdoor social distance.
Medical system	Building makeshift hospitals and international health posts. In order to ensure people’s demand for medical treatment and medicine purchase during the epidemic, some regions have implemented the policy of no referral for outpatient services.	Activated a medical emergency plan. The federal and state governments have reached an agreement to double the number of intensive care beds as soon as possible and have called for more beds in hospitals.	Developed epidemic prevention tracking software to better track populations and control the epidemic. Each state conducted nucleic acid testing in an orderly manner.
District management	Wuhan implemented the decisions and arrangements of the CPC Central Committee, closed the exit channel and put Wuhan on lockdown. Some regions have implemented epidemic control measures for transportation, and high-risk areas have been closed.	The federal and state governments had a no-contact agreement: people must stay at least 1.5 meters away from each other. Large gatherings and carnivals were prohibited in public places and private apartments.	The Delta strain was so severe that parts of Australia were under emergency lockdown and several states have issued a state lockdown. The state of Victoria and the Capital Territory have ordered lockdown as COVID-19 rebounds.
Campus measures	Delayed opening, suspension of classes, postponement of school-related work.	Schools and childcare facilities were closed and the government issued social segregation policies.	Some epidemic-related measures have been implemented, and students are studying through online classes.
Border control	Strict border management, restrictions on entry, full implementation of entry and exit personnel health declaration system, and strictly carry out entry health quarantine.	The government has tightened border controls to control the importation of cases from abroad. The European Union restricts entry to non-EU citizens.	Prime Minister Morrison has successively issued a travel ban and closed the border. Australia announced a comprehensive lockdown from March 20, 2020.
Relaxation measures	Under the joint prevention and control mechanism of The State Council, it was pointed out that scientific prevention and control should be carried out with targeted policies and at different levels. Low-risk areas require full resumption of production. The national epidemic prevention headquarters has decided to basically lift the coronavirus epidemic nationwide in early December 2022.	The German government announced a partial lifting of the lockdown to cushion the economic impact of the pandemic. It announced the resumption of some retailers and schools, but urged people to wear face masks in public. Germany has gradually relaxed in accordance with the “three-step” route, and fully liberalized in March 2022.	South Australia reopened its border to New South Wales, allowing residents of the two states to move in and out of each other without the need for a 14 days quarantine. Queensland opened its borders to parts of New South Wales. Australia has gradually relaxed its social distancing restrictions, with plans to lift them completely in July 2022.
Financial relief	Under the joint prevention and control mechanism of The State Council, four preferential tax and fee policies have been put forward to help enterprises resume work and production.	The budget for the stabilisation programme of the Federal Finance Ministry amounted to 450 billion euros.	The government has provided 100 billion Australian dollars in subsidies to ensure people’s livelihood and help them survive the epidemic.

The data came from the official websites of the three governments (15–17).

#### 3.1.2 Mitigation strategies in the United States, South Africa, and Italy

Mitigation strategies aim to keep the number of infections low through modest control measures, but could overwhelm health service capacity if COVID-19 infections increase (29). The United States, South Africa, and Italy have tended to adopt relatively lenient mitigation strategies in the fight against COVID-19. After the outbreak of COVID-19, the United States announced its first case of COVID-19 on January 21, 2020 with the number of confirmed cases exceeding 1,000 on March 10, 2020 and confirmed cases in all 50 states on March 17, 2020. The United States has adopted a strategy of “containment” and “mitigation” to respond to the epidemic through multiple channels and means, including virus testing, campus prevention and control, and social distancing. But in the early stages of the outbreak, the United States only advised the public to take precautions but not to wear masks for healthy people. Most U.S. states and territories issued stay-at-home orders and shutdowns after the government imposed quarantines and curfews as the number of confirmed cases rose. At the same time, the United States increased investment to accelerate vaccine development and vaccination schedule (30, 31). However, the epidemic has not been fully alleviated, and states across the United States have begun to relax their epidemic control measures, reopening restaurants and gradually resuming production. South Africa was the first African country to declare a state of national disaster (32). On March 5, 2020, South Africa reported its first confirmed case of COVID-19. Soon, South Africa began to seal off the country, strictly implementing the “lockdown order” throughout the country, and set the lockdown level as the most stringent level five (33). However, after May 2020, the South African government gradually lowered the lockdown level from phase 5 to Phase 1, gradually easing the quarantine measures. Since the resumption of work and production in May, the number of newly confirmed cases in South Africa has fluctuated and the epidemic has shown a rebound trend.

Italy has taken different measures to contain the spread of the virus during the COVID-19 outbreak (34). As early as January 31, 2020, the Italian government began to implement border control measures. Italy did not initially implement strict restrictions, which led to the spread of the epidemic. At this point, the government began to act, but it was too late. Italy’s large population and high population density have exacerbated the difficulties of fighting the epidemic. With the first COVID-19 case reported on February 20, 2020, the outbreak has deteriorated rapidly in Italy, which also has the highest death rate in the world. From March to May, the government divided the country into different regions and quickly adopted radical lockdown measures, followed by national lockdown measures (35). In late May, 2020, the epidemic prevention and control measures were gradually relaxed. (Examples of specific measures taken by the United States, South Africa, and Italy are shown in Table 2).

**Table 2:** Major non-drug interventions in response to COVID-19 in the United States, South Africa, and Italy.

Measure/Country	The United States	South Africa	Italy
Overall strategy	Mitigation strategy		
Government response	The U.S. government declared a national state of emergency on March 13, 2020. On April 1, US President Donald Trump approved a “state of disaster” for 30 states.	South Africa declared a state of national disaster, activated a crisis management mechanism and set up a national response command committee. South Africa’s president has announced a level 2 nationwide response to the third wave of COVID-19.	A working group on COVID-19, led by Italy’s health minister, has been set up to review the progress of the epidemic and propose measures to prevent and control it. The Italian government has declared a state of emergency.
Community policy	On March 16, 2020, the US government prepared to take any necessary measures. Consider implementing measures such as isolation and curfew in “hot spots,” but temporarily do not consider implementing them nationwide.	All social activities were banned, people were allowed to go out for food, medicine and medical treatment, and most businesses were closed. A curfew was imposed.	All activities were suspended, places of recreation closed; All sports activities were suspended and all shops except food stores and pharmacies were closed. All non-essential and non-critical production and business activities were halted.
Medical system	The first temporary hospital in New York was completed on March 28, 2020. The governor of New York State has said that there is an extreme shortage of ventilators in New York. New York had only 5,000 to 6,000 ventilators, but needed a total of 30,000, so it procured them from around the world.	A South African construction company has built an isolation facility at a hospital specifically for the novel coronavirus. The second wave of the outbreak has put a heavy strain on South Africa’s health system, with hospitals temporarily redeploying beds and staff and postponing a large number of non-emergency operations.	To ensure the safety of health workers, the Government has taken urgent action to secure more protective equipment and ensure the functioning of the health system. Makeshift hospitals have been set up, the first consisting of 15 tents with 60 beds.
District management	The Deputy Secretary of Defense has signed a memorandum of understanding on the internal travel ban.	The lockdown order was strictly enforced across the country and the lockdown level was set at level five, the most stringent.	Italy was declared a “red alert zone” and the country was put under martial law by the military.
Campus measures	Since March 8, 2020, many colleges and universities in the United States have been closed. Primary and secondary schools have suspended classes; Schools in several states began opening in August 2020.	The government decided to delay the opening of public and private schools by two weeks. Schools can resume full time learning after the cabinet issued changes to coronavirus regulations.	The Italian government decided on March 6 that all schools and universities in Italy would be closed until mid-March, and later announced that all schools would be closed for an extended period.
Border control	The US government has announced it is closing its borders with Canada and Mexico.	Borders have been closed, border controls tightened and inter-provincial travel banned.	Travel restrictions were put in place, requiring all people entering the country from Croatia, Greece, Malta and Spain to undergo virus testing.
Relaxation measures	The United States reopened its borders by lifting restrictions on foreign travelers from 33 countries and territories.	The government gradually lowered the lockdown level from phase 5 to Phase 1, relaxed quarantine measures and began to resume work and production. In June 2022, South Africa repealed its COVID-19 quarantine regulations.	Residents were allowed to move from city to city, industries began to resume work, schools resumed classes, and entry and exit travel was allowed. Since May 2022, Italy has gradually relaxed its control over the COVID-19 outbreak.
Financial relief	On March 20, 2020, the U.S. Treasury Secretary said that the Trump administration would extend this year’s tax deadline, originally set for April 15, by three months to July 15.	The South African government further improved and expanded employment-related tax incentives, and increased support for poor families, with social benefits reaching more than 10 million people.	The government offered 32 billion euros in financial aid to businesses, self-employed people, local governments and related industries affected by the pandemic.

The data came from the official websites of the three governments (18–20).

### 3.2 Vaccine measures taken by countries

The research and development of COVID-19 vaccine is a race between human beings and the virus. Different countries and research and development institutions may have different tracks, but they are heading for the same destination. Since Chinese scientists released the whole genome sequence of the novel coronavirus on Jan 11, 2020, the global research and development of a vaccine against the novel coronavirus has been in high tide. As early as March 16, 2020, a candidate COVID-19 vaccine entered clinical testing for the first time, the fastest pace in history. (Detailed information on COVID-19 vaccine development and vaccination in six countries is shown in Table 3). First, in December 2020, some 20 million health care workers and older adult people in nursing homes across the United States were among the first to receive a vaccine against COVID-19. From January to March 2021, the second batch of people who received the COVID-19 vaccine include: teachers, police and other core posts, high-risk disease patients and people over 65 years old. From April to June 2021, vaccines were available for free throughout the United States.

**Table 3:** Main vaccine interventions in six countries.

Country/Measure	Vaccine development	Vaccination
China	1. China’s Center for Disease Control and Prevention has successfully isolated the country’s first strain of the novel coronavirus. 2. A recombinant novel coronavirus vaccine has been approved for clinical trials. 3. China officially joined the COVID-19 vaccine Implementation Plan.	1. The first step was to vaccinate key groups, the second to vaccinate key groups and high-risk groups, and the third to vaccinate the general population and other groups. 2. Recent vaccination has focused on bridging the immunity gap between different target populations.
Germany	1. Germany approved a clinical trial of a novel coronavirus vaccine candidate made by German biotech company BioNTech. 2. In the first phase of the clinical trial, 200 volunteers between the ages of 18 and 55 were given several different vaccines.	1. December 2020–April 2021: vaccinated very high-risk populations. 2. May–July 2021: vaccinated high-risk populations. 3. July–August 2021: Vaccinated moderate-risk groups. 4. August–November 2021: vaccinated relatively high-risk and relatively low-risk populations. 5. December 2021: vaccinated low-risk populations.
Australia	1. A proprietary technology was used in a bid to fast-track production of a vaccine against the novel coronavirus. 2. Within 3 weeks, the team had the first vaccine candidate in the lab.	1. The first doses were given to front-line workers and people who had been in contact with international travellers. 2. It announced plans to complete a mass vaccination programme for its 25 million citizens by the end of 2021.
The United States	1. The first US vaccine against COVID-19 was injected into people in a human trial involving 45 volunteers. 2. Pfizer announced the simultaneous launch of its mRNA vaccine, developed in collaboration with BioNTech.	1. December 2020: Health care workers and older adult people in nursing homes. 2. January–March 2021: Core job groups such as teachers and police officers, patients with high-risk diseases and seniors over 65 years old. 3. April–June 2021: Free vaccine available throughout the United States.
South Africa	1. Afrigen, a South African biotechnology company, has successfully developed and produced the continent’s first mRNA vaccine against COVID-19.	1. The plan was to launch phase 2 and Phase 3 vaccinations on May 17 and October 17. 2. The plan was to vaccinate two-thirds of the population by the end of 2021.
Italy	1. In 2020, the first phase of a novel coronavirus vaccine was successfully tested in Italy, where antibodies produced in mice proved effective. 2. The National Institute of Infectious Diseases recruited volunteers for a clinical trial of the vaccine.	1. Italy officially started the COVID-19 vaccination campaign on December 27, 2020, and people in Italy can receive free vaccines according to the order of vaccination published by the Ministry of Health. 2. The vaccination of persons in Italy was phased in four phases. The first phase was from January to March, the second from April to June, the third from July to September, and the fourth from October to December.

The data came from the official websites of the six governments (15–20).

### 3.3 Analysis of epidemic prevention and control effect in six countries

#### 3.3.1 Basic information and epidemic situation in six countries

The basic characteristics of the six countries are shown in Table 4. Australia is the lowest population density of the six countries, which may reduce the difficulty of prevention and control to some extent, while Germany is the highest population density of the six countries. China and South Africa have relatively small populations over the age of 65. Of the six countries, China has the fewest cumulative confirmed cases per million people and cumulative deaths per million people. Of the six countries, China had the highest tightening index, while Australia and South Africa had the lowest. Germany has the highest number of hospital beds per 1,000 people, significantly higher than any other country. As the coronavirus epidemic has spread, governments have embraced a variety of non-drug interventions and issued a series of policy documents. With the changes in the trend of the epidemic and the needs of economic and social development, each country adjusted its prevention and control strategy based on its own national conditions.

**Table 4:** Comparison of basic characteristics of six countries (as at 31 December 2022).

Indicator/Country	China	Germany	Australia	The United States	South Africa	Italy
Population	1,425,887,360	83,369,840	26,177,410	338,289,856	59,893,884	59,037,472
Population density (per square kilometer)	147.67	237.02	3.20	35.61	46.75	205.86
Aged 65 older	10.64	21.45	15.50	15.41	5.34	23.02
GDP per capita	15308.71	45229.25	44648.71	54225.45	12294.88	35220.08
Total cases per million	1371.83	448242.03	425240.96	297864.18	67606.27	425894.00
Total deaths per million	3.68	1936.73	651.40	3230.47	1712.50	3127.54
Stringency index	47.69	14.81	11.11	28.72	11.11	21.99
Hospital beds per thousand	4.34	8.00	3.84	2.77	2.32	3.18

The data came from our world in data (13).

**Figure 1:**
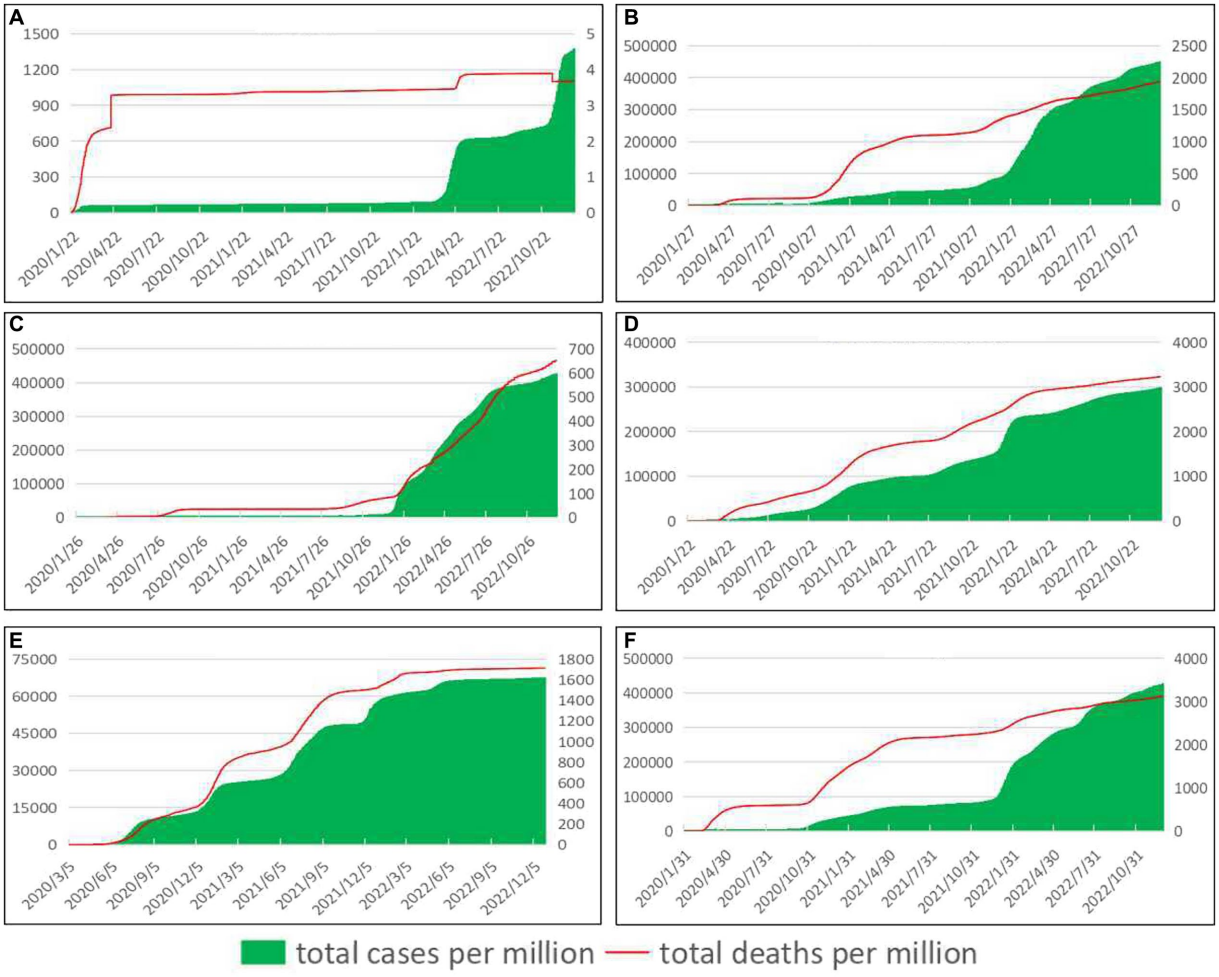
Trends in total cases and deaths per million people. See primary axis (left) for “total cases per million people” and secondary axis (right) for “total deaths per million people” (The data came from our world in data (13)).

Figure 1 (Trends in total cases and deaths per million people) shows that the six countries have different epidemic prevention and control effects. In China, the cumulative number of confirmed cases per million people increased slowly and at a low level in the early stage, and there were two rising peaks in the later stage. The cumulative death cases per million people rose twice at the early stage of the epidemic, and then the growth rate was very slow. In Germany, the cumulative confirmed cases per million people and cumulative deaths per million people grew relatively slowly on the whole, but there was a peak of growth, respectively. In the early stage of the epidemic, the epidemic in Australia was well controlled, but since 2022, the epidemic has deteriorated sharply, with the cumulative number of confirmed cases per million people and the cumulative number of deaths per million people, respectively, showing a peak increase. The epidemic is growing rapidly in the United States, with the cumulative number of confirmed cases per million and the cumulative number of deaths per million continuing to increase. In South Africa, the cumulative number of confirmed cases and deaths per million people increased rapidly in the early period and experienced several growth peaks, but the growth slowed down in the later period. In Italy, the cumulative number of confirmed cases per million people increased rapidly, with one peak, and the cumulative number of deaths per million people showed a stepped increase trend, with three peaks. As of December 31, 2022, the cumulative number of confirmed cases per million people in China, Germany, Australia, the United States, South Africa and Italy was 1371.83, 448242.03, 425240.96, 297864.18, 67606.27 and 425894.00, respectively. The cumulative death cases per million population were 3.68, 1936.73, 651.40, 3230.47, 1712.50 and 3127.54.

#### 3.3.2 The three principles of epidemic prevention in six countries

##### 3.3.2.1 Control the source of infection

**Figure 2:**
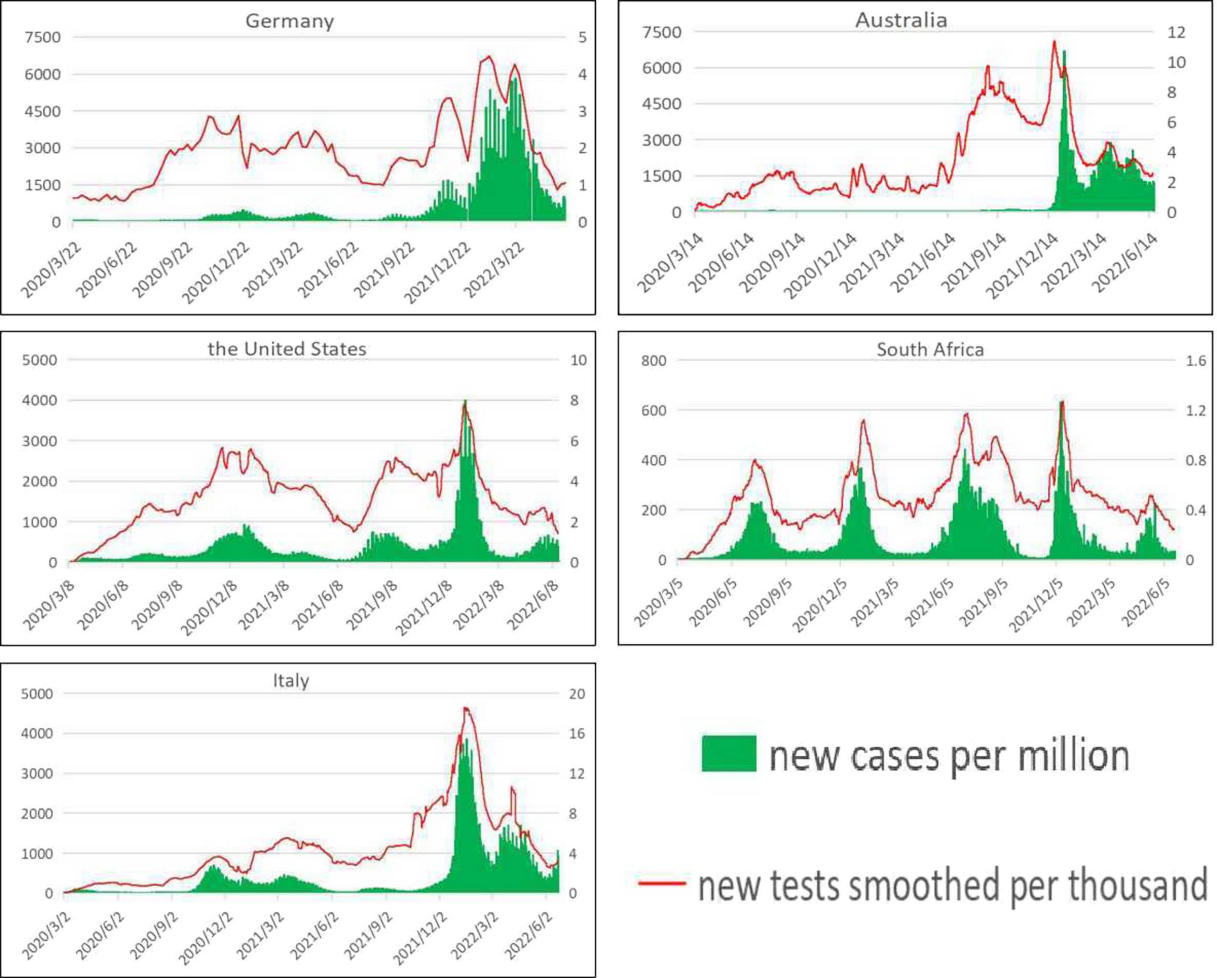
Trends in daily new cases per million people and daily tests per thousand people. See primary axis (left) for “daily new cases per million people” and secondary axis (right) for “daily tests per thousand people” (The data came from our world in data (13)).

Since the outbreak of the novel coronavirus, countries have tested their citizens in order to keep track of the situation. As can be seen from Figure 2 (Trends in daily new cases per million people and daily tests per thousand people), when the number of newly confirmed cases increased, countries intensified their COVID-19 testing efforts to control the further epidemic to some extent, and the number of newly confirmed cases showed a downward trend after the number of each test reached its peak. Germany, the United States, South Africa, and Italy are particularly visible. In addition, a reduction in the number of daily tests may also lead to a sharp increase in the number of daily new confirmed cases in the future. For example, South Africa increased the number of tests when the epidemic was severe, and then relaxed after getting the epidemic slightly under control, leading to a rebound of the epidemic, and thus multiple peaks. During the epidemic period, in order to control and manage the source of infection in a timely manner and achieve early detection, isolation and treatment, multiple rounds of large-scale testing were carried out in various regions of China, and regular testing was flexibly arranged in accordance with the development of the epidemic situation. Since the number of daily tests in China is not reported continuously, there is no complete official authoritative data on daily tests per thousand people. The analysis of discontinuous data may cause errors in the results, so it is not shown in Figure 2.

##### 3.3.2.2 Cut off transmission routes

**Figure 3:**
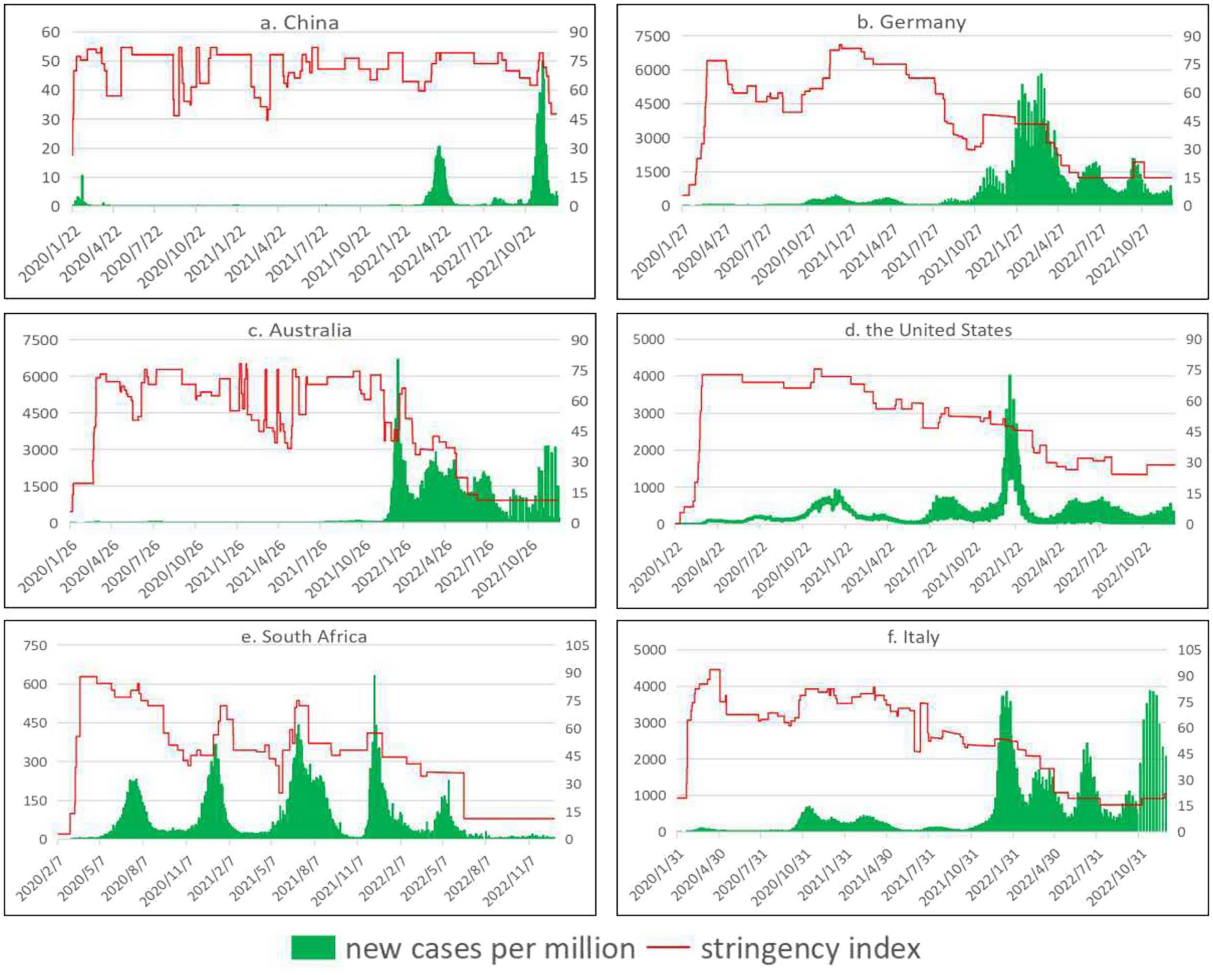
Trends in daily new cases per million people and stringency index. See primary axis (left) for “daily new cases per million people” and secondary axis (right) for “stringency index” (The data came from our world in data (13)).

Novel coronavirus pneumonia is a kind of acute infectious pneumonia, its infectivity is relatively strong, most will be transmitted through respiratory droplets. As a result, countries have adopted a series of measures to block transmission routes. As can be seen from Figure 3 (Trends in daily new cases per million people and stringency index), since 2020, countries have introduced a series of epidemic prevention and control policies when the epidemic becomes more serious, but the degree of stringency and effectiveness of prevention and control policies vary from country to country. In general, in the early stage of the epidemic, countries adopted stricter epidemic prevention and control policies with a higher stringency index. In the later period, epidemic prevention and control was gradually relaxed, and the level of stringency index was lowered. In addition, when the pandemic situation is severe, the stringency index of countries increases; as the situation of the epidemic eases, the stringency index of countries shows a downward trend, but as the epidemic prevention and control is relaxed, the epidemic will rebound, such as in the United States.

##### 3.3.2.3 Protect vulnerable populations

**Figure 4:**
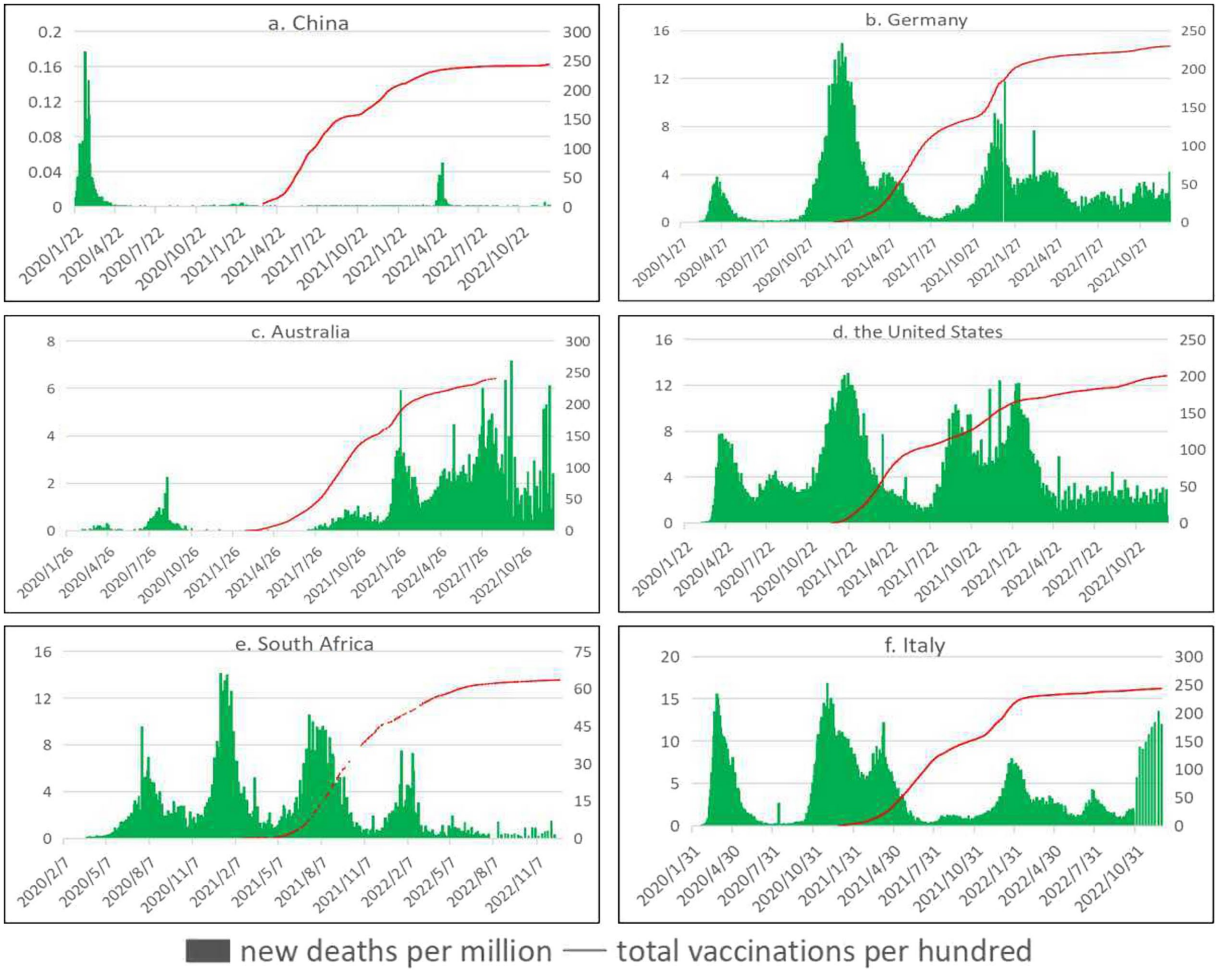
Trends in daily new deaths per million people and total vaccinations per hundred people. See primary axis (left) for “daily new deaths per million people” and secondary axis (right) for “total vaccinations per hundred people” (The data came from our world in data (13)).

Since the outbreak of COVID-19, countries have actively developed vaccines and carried out continuous vaccination. As can be seen from Figure 4 (Trends in daily new deaths per million people and total vaccinations per hundred people), the number of daily new deaths per million people in China, Germany, and South Africa showed a downward trend with the increase of the number of vaccines administered per 100 people. Even if there was a peak, the number of daily new deaths per million people was moderate compared with the early stage of the epidemic. In Australia, the United States, and Italy, daily new deaths per million people continued to peak after vaccination, and the outbreak has eased, but not significantly. According to the latest statistical data in December 2022, the number of people fully vaccinated per 100 people in China, Germany, Australia, the United States, South Africa, and Italy is 89.35, 76.23, 82.72, 69.09, 35.13, and 81.26, respectively. Germany, Australia, the United States, and Italy had a high rate of full vaccination, but also saw a peak in the number of daily new deaths. South Africa has a low rate of full vaccination, but it also saw a decline in the number of daily new deaths in the later stages of the epidemic.

## 4 Discussion

### 4.1 Containment strategies and mitigation strategies

Since the outbreak of COVID-19 as a public health emergency, countries have taken a series of measures and issued a series of policy documents in various aspects. For example, home quarantine, shutdown of production and schools, emergency response mechanism, strict border control, vaccine clinical trials, etc. In addition, some scholars have explored how transmission can decline after disease detection and implementation of combined non-drug measures, based on the analysis of the early epidemiological characteristics of the COVID-19 outbreak (36). The policies and measures adopted by various countries in response to the COVID-19 epidemic have their own characteristics, strengths and weaknesses, and the effectiveness of epidemic prevention is also significantly different. China, which adopted a strict containment strategy, initially contained the spread of the epidemic in more than a month, controlled the daily new cases within a single digit in about 2 months, and achieved decisive results in the defense battle in Wuhan and Hubei in about 3 months. However, the United States, which adopted mitigation strategies, once had the highest cumulative number of confirmed cases and cumulative number of deaths in the world (37). In addition, some scholars have conducted in-depth analysis of the measures taken during the epidemic in many countries, and believe that non-drug interventions can effectively level the epidemic curve, and rapid and strict comprehensive containment strategies are successful measures to control the epidemic, especially in the absence of vaccines and effective therapies (38–40). In view of this, countries should be able to make prompt decisions when responding to public health emergencies, have enough sensitivity, and adopt strict containment strategies in a timely manner to reduce the risk of explosive spread of the epidemic.

### 4.2 Control the source of infection

Infectious diseases are diseases caused by various pathogens that can be transmitted from person to person. In the prevention and treatment of infectious diseases, it is necessary to control the source of infection, such as early detection and treatment, and observation of close contacts. Early detection of the novel coronavirus is extremely important to contain the spread of the pandemic. It can grasp the development of the epidemic to a certain extent, track cases in a timely manner, and intervene in close contacts. Germany and Hong Kong have rapidly developed the WHO-approved PCR test, which has become the standard around the world (except in the United States). The Australian government had allocated more than a $750 million to ramp up testing, which in the early months of the outbreak was among the highest in the world in testing per 1,000 people. Extensive and frequent testing is also critical to the containment of COVID-19 in the United States (41). Combined, the countries showed a decline in the number of daily new cases per million people in the coming months after the increased testing. With the different situation of the development of the epidemic, countries have also made adjustments to the cost of testing. For example, whether nucleic acid testing is free in China depends on the identity of the person tested and the local disease control policy. Generally, for personnel who must be tested, the cost of nucleic acid testing shall be borne by the state and local governments, while for personnel who voluntarily test, the cost of nucleic acid testing shall be borne by units or individuals. Therefore, when dealing with COVID-19 and other similar public health emergencies, we should actively carry out case detection, and strive to achieve early detection and isolation, so as to reduce the probability of human to human transmission.

### 4.3 Cut off transmission routes

Infectious diseases are spread by respiratory droplets, blood transmission, contact transmission and so on. After an outbreak of an infectious disease, disinfection and isolation can be carried out. For example, wear a mask, keep the air circulating at home, and sterilize the air when necessary. Some researchers have also designed and evaluated various strategies to increase people’s wearing of masks, and have assessed the impact of community wearing of masks on COVID-19 infection rates (42). Some countries like China implemented strict policies at the early stage of the epidemic. As the epidemic eased, countries gradually relaxed their vigilance and relaxed quarantine and mask measures, leading to varying degrees of rebound of the epidemic. For example, around Independence Day in the United States in 2020, people held large outdoor parties to celebrate Independence Day. Crowds were crowded and many people did not wear masks, leading to an increasing number of cluster infections, and the number of people infected with the novel coronavirus continued to soar in about 40 of the 50 states. Therefore, strict government policies, especially quarantine policies, are essential for epidemic prevention and control, especially in the early stages without vaccine assistance. However, it is worth noting that the implementation time of lockdown policies in many countries has also greatly affected the effectiveness of epidemic prevention and control. In early December 2019, unexplained infections were first detected in Wuhan, Hubei Province, China. The Wuhan lockdown, which took place on January 23, 2020, was one of the largest and most widespread in China’s history, and played an important role in the country’s ability to quickly contain the spread of the disease. South Africa’s Minister of Health held a press conference on 5 March 2020 to confirm the first confirmed case of novel coronavirus pneumonia in South Africa. In order to contain the rapid spread of the epidemic, the National COVID-19 Command Committee decided to implement a nationwide lockdown from March 26 local time. On the evening of February 20, 2020, a hospital detected Italy’s first indigenous confirmed case, known as Patient 1. Italy’s prime minister has announced a nationwide lockdown from March 10, 2020. During this time, Italy has seen an explosion in confirmed cases. Comparing the time of the first confirmed case and the time of strict lockdown measures in these countries, we can find that the response measures in countries are relatively delayed. Although these efforts are commendable, relatively delayed policies will weaken the effect. Major infectious diseases and other sudden public health emergencies pose a threat to human health. Countries should implement strict policy measures in the face of similar public health emergencies, restrict personnel contact from multiple aspects, cut off the transmission routes of the virus, and strive to control the epidemic in a short period of time. At the same time, the public should also actively cooperate with government policies, wear masks, actively isolate at home, maintain social distance, work together, and overcome difficulties together.

### 4.4 Protect vulnerable populations

The COVID-19 epidemic has gradually spread around the world, with a rapid increase in confirmed cases, increasing the burden on medical systems and shortages of health supplies in many countries, and prompting countries to take response measures. Germany’s Federal Ministry of Health has focused on purchasing protective equipment for clinics and hospitals, doubling the number of intensive care beds in the short term. In March 2020, a US Navy hospital ship docked at SAN Pedro Pier in Los Angeles to provide 1,000 beds for California, which was severely affected by the epidemic, and ease the burden on onshore hospitals. On March 27, 2020, the governor of New York State announced the completion of the first temporary hospital in Manhattan, New York City, to adapt to the COVID-19 outbreak. At the same time, countries are also actively carrying out vaccine research and development and vaccination. When the human body resistance is relatively poor, the risk of infectious diseases is also greater. The older adult, children, pregnant women and other groups have poor resistance, and are susceptible to infectious diseases. These people can then receive the appropriate vaccine, improve the body’s immune mechanism. Vaccination can prevent the infection of novel coronavirus or Omicron, and reduce the incidence of novel coronavirus infection. Vaccination of novel coronavirus vaccine is of great significance to strengthen the immune barrier of the population and stop the epidemic of novel coronavirus pneumonia. Some scholars have confirmed the effectiveness of BNT162b2 vaccine against COVID-19 infection in their studies and some research results have provided support for high effectiveness of BNT162b2 against hospital admissions up until around 6 months after being fully vaccinated (43, 44). Adherence to COVID-19 preventive measures and appropriate population vaccination levels are undoubtedly important means to control the outbreak (45). Of the six countries, China has the highest number of fully vaccinated people per 100 and the fewest cumulative deaths. South Africa also has low vaccination rates and should use available COVID-19 vaccines (46). It is worth noting that incomplete vaccine coverage, combined with continued community transmission, has facilitated the emergence of mutated strains. The protective effectiveness of the vaccine wanes to some extent over time, so when the mutant strain appears, countries bounce back due to its greater transmissibility and shorter incubation period. To sum up, countries should actively support vaccine research and development and vaccination when responding to public health emergencies such as the COVID-19 outbreak. People in all countries should pay attention to physical fitness and actively cooperate with the vaccination work. However, it is important to note that vaccination measures cannot replace non-drug interventions, and the two complement each other to have a greater impact.

### 4.5 Limitations

The study also has the following limitations. The stringency index used in this article does not measure or imply the adequacy or effectiveness of a country’s response. A higher score does not necessarily mean a better response from a country. For example, it may be that some countries have a high stringency index, but there are large election campaigns or large rallies in the meantime, and this does not reflect it. In ecological research, ecological fallacy is the most important shortcoming of this kind of research, which is because ecological research is made up of individuals in different situations, the group is the unit of observation and analysis, and the existence of confounding factors and other reasons cause the research results are not consistent with the real situation. For this reason, it is difficult to avoid ecological fallacy in ecological research in general. In view of the characteristics and limitations of ecological research, this study focuses on the research purpose as much as possible and sets only one research question. At the same time, more variables are included in the process of analyzing the problem and testing the hypothesis to reduce errors. In addition, due to statistical differences between countries, it is not possible to find all the continuous data needed for the study, resulting in many analysis limitations. Finally, when speculating on the research results, try to compare with other non-ecological research results, and combine the professional knowledge of the research problem to make a comprehensive analysis and judgment. In addition, factors such as population, culture, geography and economic level have complicated impacts on the development and prevention and control of the epidemic, and further in-depth research is needed.

## 5 Conclusion

This study analyzed the effect of non-drug intervention measures and vaccine coverage on COVID-19 prevention and control through ecological comparative study on COVID-19 in six countries. Based on the three principles for the prevention and control of epidemiological infectious diseases, the current situation of epidemic prevention and control in each country and the effect of prevention and control measures were analyzed in detail. Studies have confirmed that the implementation of non-drug interventions and vaccination will greatly affect the effectiveness of epidemic prevention and control. In the early stage of the epidemic, in the absence of effective vaccine support, non-drug interventions are an important means to deal with the epidemic, and a rapid and strict comprehensive containment strategy is an important measure to control the rapid spread of the epidemic. Vaccine development and vaccination is an important part of the prevention and control of COVID-19. However, the coverage rate of vaccines in some countries is still low, so it is still necessary to vigorously promote the vaccination of COVID-19 vaccines and booster shots. Vaccines cannot replace non-drug interventions, and when vaccine coverage is insufficient to establish a solid population immunity barrier, premature elimination of non-drug interventions is highly likely to lose the previous prevention and control achievements, leading to the rebound of the epidemic. Therefore, vaccination alone cannot completely stop the outbreak and needs to be combined with non-drug interventions. Each community should do a good job in health education, vaccination, and disease detection to improve the health literacy and disease prevention ability of residents. All countries should actively participate in international cooperation, review the experience of responding to public health emergencies in COVID-19 prevention and control, and learn from the successful experience of other countries to make up for their weaknesses. Countries also need to improve their public health emergency response mechanisms and be able to respond quickly to public health emergencies. In view of the limitations and shortcomings of this study, the team will use more accurate data and more abundant indicators in the future to conduct in-depth research on global epidemic prevention and control, and continue to explore effective measures to deal with public health emergencies.

## Data availability statement

Publicly available datasets were analyzed in this study. This data can be found at: https://github.com/owid/covid-19-data/tree/master/public/data.

## Author contributions

ML: Data curation, Funding acquisition, Investigation, Methodology, Project administration, Resources, Supervision, Writing – original draft, Writing – review & editing. LS: Data curation, Project administration, Resources, Writing – review & editing. MY: Data curation, Writing – review & editing. JJ: Data curation, Writing – review & editing. JY: Data curation, Supervision, Writing – review & editing. MM: Data curation, Supervision, Writing – review & editing. WX: Data curation, Investigation, Writing – review & editing. GS: Data curation, Funding acquisition, Methodology, Resources, Supervision, Writing – original draft, Writing – review & editing.
